# Functional Substrate Mapping of the Right Atrium: A Novel Method to Identify Critical Isthmus of Re-entry in Atrial Tachycardia

**DOI:** 10.19102/icrm.2025.16073

**Published:** 2025-07-15

**Authors:** Hikmet Yorgun, Cem Çöteli, Gül Sinem Kılıç, Samuray Zekeriyeyev, Muhammet Dural, Kudret Aytemir

**Affiliations:** 1 Department of Cardiology, Faculty of Medicine, Hacettepe University, Ankara, Turkey; 2 Department of Cardiology, Cardiovascular Research Institute Maastricht (CARIM), Maastricht University Medical Center, Maastricht, The Netherlands; 3 Department of Cardiology, Faculty of Medicine, Eskişehir Osmangazi University, Eskişehir, Turkey

**Keywords:** Atrial late activation mapping, atrial tachycardia, fragmented electrogram, functional substrate mapping, re-entry

## Abstract

Right atrial tachycardia (AT) is a frequent rhythm disorder in patients with atrial scar mainly due to surgical incisions or congenital heart diseases. Despite the mounting evidence about AT mechanisms and types, data are scarce regarding the conduction properties as well as the functional characteristics of the atrial substrate during sinus rhythm, which plays a role in the maintenance of tachycardia. We sought to evaluate the relationship between the functional substrate mapping (FSM) characteristics of the right atrium (RA) and the critical isthmus (CI) of re-entrant ATs in patients with underlying atrial scar. Patients with a history of right AT who underwent catheter ablation with three-dimensional mapping were retrospectively enrolled. A voltage map and isochronal late activation map were created during the sinus/paced rhythm using multielectrode catheters to detect deceleration zones (DZs). Subsequently, AT was induced with programmed stimulation, and activation mapping was performed to detect the CI of the tachycardia. Atrial tachyarrhythmia (ATa) recurrence was defined as the detection of atrial fibrillation or AT (≥30 s) during follow-up. A total of 24 patients (mean age, 46 ± 15 years; 13 [54%] women) with right AT were included. A total of 36 ATs were mapped (16 [44.4%] localized re-entry, 20 [55.6%] macro–re-entry). Atrial low-voltage zones composed 23.3% ± 13.0% of the total RA. The mean values of bipolar voltage, electrogram duration, and conduction velocity during sinus rhythm corresponding to the CI of ATs were 0.18 ± 0.10 mV, 121.7 ± 29.4 ms, and 0.06 ± 0.04 m/s, respectively. The total number of DZs per chamber was 1.1 ± 0.3, with all being located in the low-voltage zone (<0.5 mV) detected by high-density mapping. All CIs of non-cavotricuspid isthmus (CTI)-dependent re-entry were co-localized with DZs detected during FSM. The positive predictive value of DZs to detect the CI of inducible ATs was 80.8%. During a mean follow-up of 11.7 ± 8.1 months, freedom from atrial tachyarrhythmias was 87.5%. Although CTI-dependent macro–re-entry is the most common mechanism in patients with RA scar, our findings demonstrated the relevance of FSM to predict non–CTI-dependent ATs. Conduction slowing manifested as DZs with continuous-fragmented signal morphology may guide ablation strategy tailoring in the case of underlying RA scar.

## Introduction

Right atrial tachycardia (AT) is a frequent rhythm disorder seen especially in patients with a structurally remodeled atrium as a result of atrial dilation, surgical incisions, or congenital heart disease. Cavotricuspid isthmus (CTI)-dependent macro–re-entry is the most commonly reported mechanism; however, non–CTI-dependent ATs are also frequent, especially in the case of an underlying atrial scar. Although activation mapping and entrainment maneuvers can provide valuable information about the mechanism of clinical tachycardia, not all patients are admitted to the electrophysiology laboratory during AT. In such cases, voltage mapping during sinus rhythm can give important data about the atrial substrate harboring the critical isthmus (CI) of re-entrant AT. Nevertheless, the functional relevance of a dense scar or borderline low-voltage regions to estimate the CI of re-entry has yet to be defined.

The effect of surgical incisions or fibrosis on conduction properties leading to either CTI-dependent or non–CTI-dependent macro–re-entry has long been known.^1,2^ However, beyond voltage analysis, functional substrate mapping (FSM) characteristics of the right atrium (RA) have not been evaluated before. The use of FSM during sinus rhythm by means of isochronal late activation mapping (ILAM) to define critical sites of re-entry in the case of ventricular tachycardia has already been defined.^3,4^ Recently, several studies evaluated the utility of FSM in the atrium to predict critical sites of the re-entrant circuit.^5–7^ Furthermore, using high-density mapping, we recently reported the correlation between critical sites of re-entry in the left atrium and deceleration zones (DZs) during sinus/paced rhythm, which highlighted the significance of functional atrial substrate to predict the CI of AT.^8^

In this study, we sought to evaluate the FSM characteristics of RA by means of bipolar voltage, electrogram (EGM) signal duration, and conduction velocity (CV) using atrial late activation mapping and their correlation with critical sites of re-entry in patients with atrial low-voltage zones. Furthermore, the acute and mid-term outcomes of AT ablation were also presented.

## Methods

### Study population

In this retrospective analysis, we included all patients with scar-related right AT who underwent three-dimensional electroanatomical mapping during sinus rhythm between February 2021 and July 2023. Scar-related AT was defined as tachycardia in the case of atrial low-voltage areas (<0.5 mV). In all patients, FSM was performed at the beginning of the procedure during sinus/paced rhythm before the induction of AT. Baseline demographic characteristics and past medical history were taken from the patient’s hospital records. The study was approved by the local ethics committee.

### Preprocedural management

Before the procedure, transthoracic and transesophageal echocardiography was also performed to evaluate right and left ventricular functions and valvular diseases to exclude atrial thrombus. Computed tomography was performed to evaluate the anatomy in congenital heart diseases when needed. Anti-arrhythmic drugs (AADs) were discontinued at least five half-lives before the procedure, except for amiodarone. Novel oral anticoagulants were discontinued 24 h before the procedure, and we proceeded with the procedure when the international normalized ratio level was <2.5 in patients receiving warfarin.

### Electrophysiological study

All procedures were performed under either conscious sedation or general anesthesia. A 6-Fr steerable diagnostic catheter was placed into the coronary sinus (CS) and fixed during the procedure. Three-dimensional electroanatomical mapping was performed using either CARTO™ 3 (Biosense Webster, Diamond Bar, CA, USA) or EnSite™ Precision/EnSite™ X (Abbott, Chicago, IL, USA) with high-density multipolar catheters (PentaRay^®^ [Biosense Webster] and Advisor™ HD Grid [Abbott]). The details of atrial FSM have been previously described elsewhere.^8^ Briefly, ILAM was completed during either sinus or paced rhythm by automatic/manual annotation at the offset of the latest atrial deflections from the baseline. The atrial activation window was set between the onset of the earliest and offset of the latest local EGM recorded by a high-density mapping catheter in the RA. Afterward, total activation was divided into eight equally distributed isochrones. Regions with isochronal crowding, which were also defined as DZs, were the areas with ≥3 isochronal colors within a 1-cm radius. CV was calculated using two points in the same line of the propagation vector both for DZs and CI during sinus/paced rhythm and AT, respectively.^9^ Additionally, double potentials were tagged in different colors and annotated as follows: (1) first deflection of the double potential was annotated if it was in the early area and (2) second deflection of the double potential was annotated if it was in the later activated region. A fragmented EGM was defined as ≥4 deflections in an atrial bipolar electrogram from the isoelectric line and tagged in a different color.^10^ Voltage settings for substrate mapping were as follows: low-voltage zone ≤ 0.5 mV and normal > 0.5 mV.

After completion of FSM, AT was induced with programmed stimulation either from a CS catheter or a catheter placed on a DZ of the ILAM. After having a stable CS position as an atrial reference, the window of interest was set in order to identify the re-entrant circuit, and activation mapping in the whole chamber was performed.^11^ Briefly, macro–re-entry was defined as an activation sequence covering >90% of the tachycardia cycle length (TCL) with a circular type of activation pattern.^12^ Focal AT was defined when a distinct source of a radial activation pattern with presystolic potentials was identified in the activation map. Localized re-entry was defined in the case of continuous or fragmented potentials spanning approximately 50% of the TCL around a diameter of <2 cm.^13^ The CI of the tachycardia was defined as the site with the slowest conduction based on a propagation map and CV analysis. Entrainment maneuvers were performed to define the CI if the activation or propagation map was unequivocal (concealed entrainment and a return cycle length of <30 ms).^14^

**Supplementary Figure S1:**
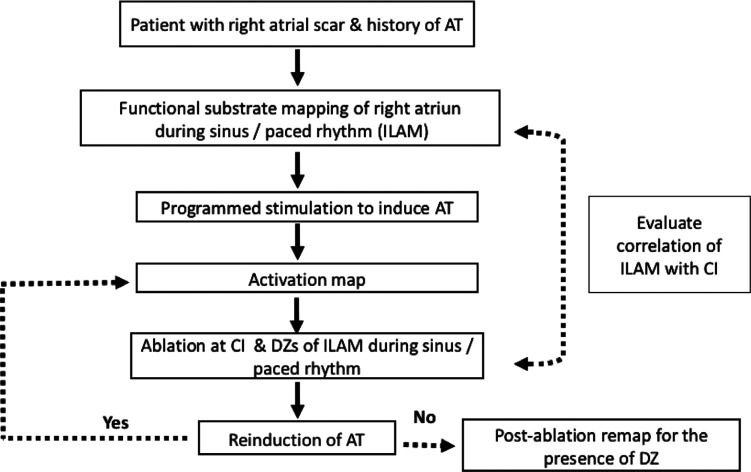
Flowchart of study demonstrating inclusion criteria as well as mapping and ablation strategy. *Abbreviations:* AT, atrial tachycardia; CI, critical isthmus; CV, conduction velocity; DZ, deceleration zone; ILAM, isochronal late activation mapping.

Signal duration and bipolar voltage amplitude of EGMs at the CI as well as CV were recorded during both sinus rhythm and AT. The correlation between low-voltage areas and CV as well as DZs of the ILAM was assessed in each patient. The flowchart of the procedure is shown in **Supplementary Figure S1**.

### Ablation approach

Phrenic nerve stimulation was performed using an ablation catheter, and nerve trajectory was tagged if the ablation target was in the lateral wall. Following definition of the re-entry circuit, radiofrequency ablation was performed using an irrigated-tip contact force–enabled ablation catheter with an energy setting of 25–40 W (contact force > 10 g, temperature limit < 42°C). Successful ablation was defined as either termination of or a change in AT. Programmed atrial stimulation was performed after AT termination, and subsequent activation maps were created in the case of inducibility. For CTI-dependent macro–re-entry, linear ablation lesions were created between the tricuspid annulus and the inferior vena cava, and a bidirectional block across each line was demonstrated. In the case of non–CTI-dependent re-entry (lateral, septal, and posterior walls), linear lesion was created in the corresponding wall between the low-voltage zone and the inferior vena cava. Focal ablation was performed in the case of localized re-entries. DZs and fragmented EGMs obtained during sinus/paced rhythm were also included in the ablation sets. After the completion of ablation lesions, substrate mapping during sinus/paced rhythm was repeated to confirm the elimination of DZs, and additional ablation was done in the case of residual fragmented EGMs. Non-inducibility of tachycardia and abolition of abnormal EGMs and DZs were considered as the procedural primary endpoint. CTI ablation was added to the ablation lesion sets in all patients if it was not blocked in the previous procedure.

### Postprocedural care and follow-up

All patients were monitored at the coronary care unit for at least 24 h after ablation. Oral anticoagulation was started the day after the procedure. Routine follow-up visits were scheduled for 1, 3, and 12 months and every 12 months thereafter or earlier when needed. Atrial tachyarrhythmia (ATa) was defined as the detection of atrial fibrillation (AF) or AT (≥30 s). A 24-h Holter monitoring session was scheduled for 3 and 12 months after the procedure and yearly thereafter or if the patient had complaints compatible with AT. Patients remained on the AAD regimen that was prescribed before their ablation in the first 3 months after ablation, with further continuation of AADs left at the discretion of the physician.

### Statistical analysis

All statistical analysis was performed using the SPSS statistical software version 22.0 (IBM Corp., Armonk, NY, USA). Descriptive and categorical variables were presented as counts and percentages. Normal distribution assumption was examined with a detrended Q–Q plot and Kolmogorov–Smirnov test. The Kaplan–Meier curve was used to demonstrate freedom from ATa recurrence during the follow-up period. The continuous data with normal distribution are expressed as mean ± standard deviation values, while data without normal distribution are expressed as median and interquartile range values. Receiver operating characteristic curve analysis was performed to evaluate the optimal cut-off value of the low-voltage area ratio for the prediction of ATa. A two-tailed *P* value of <.05 was considered to indicate statistical significance.

## Results

### Baseline characteristics

A total of 24 patients (mean age, 46 ± 15 years; 13 [54%] women) were included in the analysis. Twelve (50%) patients had a history of either surgical or catheter ablation for AF/right AT (2 [8%] pulmonary vein isolation, 10 [40%] right atrial ablation). Twenty-one (87.5%) patients had a prior history of cardiac surgery, while 18 (75%) had congenital heart disease. The detailed baseline demographic and clinical characteristics of the study population are demonstrated in **Table 1**.

**Table 1:** Baseline Characteristics of the Study Population (n = 24)

Age, years	46 ± 15
Sex, female, n (%)	13 (54%)
Cardiovascular risk factors, n (%)	
Hypertension	6 (25%)
CHA_2_DS_2_-VASc_2_, median, 25^th^–75^th^ percentile	1.00 (0.00–1.75)
Medications before ablation, n (%)	
β-Blockers	16 (66.7%)
Calcium channel blockers	4 (16.7%)
AADs	10 (40%)
Flecainide	2 (8.3%)
Amiodarone	5 (20.8%)
Propafenone	2 (8.3%)
Sotalol	1 (4.2%)
Echocardiographic parameters	
LA diameter, mm	40.7 ± 5.2
LVEDD, mm	47.0 ± 3.9
LVEF, %	57.2% ± 5.3%
RVEDD, mm	31.2 ± 10.1
sPAP, mmHg	43.4 ± 10.7
Mitral regurgitation, n (%)	
Mild	17 (70.8%)
Moderate to severe	7 (29.2%)
Tricuspid regurgitation, n (%)	
Mild	5 (20.8%)
Moderate to severe	17 (70.8%)
Anticoagulant drug, n (%)	
Apixaban	5 (20.8%)
Edoxaban	1 (4.2%)
Rivaroxaban	4 (16.7%)
Varfarine	5 (20.8%)
Previous ATa/AF catheter ablation, n (%)	
Cryoablation	1 (4.2%)
CTI ablation	6 (24%)
RA, lateral	4 (16.7%)
Cardiac surgery, n (%)	
MVR	4 (16.7%)
Tricuspid valve annuloplasty	2 (8.3%)
AVR	2 (8.3%)
PVR	2 (8.3%)
Fontan operation	2 (8.3%)
Surgical repair of TOF	5 (20.8%)
Repair of double-outlet right ventricle with conduit	1 (4%)
Surgical closure of ASD	9 (37.5%)
Surgical closure of VSD	2 (8.3%)
Anomalous pulmonary venous drainage repair	1 (4%)

*Abbreviations:* AAD, anti-arrhythmic drug; AF, atrial fibrillation; ASD, atrial septal defect; ATa, atrial tachycardia; AVR, aortic valve replacement; LA, left atrium; LVEDD, left ventricular end-diastolic diameter; LVEF, left ventricular ejection fraction; MVR, mitral valve replacement; PVR, pulmonary valve replacement; RA, right atrium; RF, radiofrequency; RVEDD, right ventricular end-diastolic diameter; sPAP, systolic pulmonary artery pressure; TOF, tetralogy of Fallot; VSD, ventricular septal defect.

### Electrophysiological characteristics during sinus/paced atrial rhythm

In all patients, FSM was performed before induction of the tachycardia during sinus rhythm (75%), distal CS pacing (20.8%), or high right atrial pacing (4.2%). The median number of mapped points during FSM was 2157 (340–3871). Atrial low-voltage zones composed 23.3% ± 13.0% of the total RA. The median low-voltage area ratio was calculated as 23.6% (8.6%–45.2%) in the posterior wall and 23.6% (9.6%–53.2%) in the lateral wall. The mean value of EGM duration and bipolar voltage at DZs during sinus rhythm was 121.7 ± 29.4 ms and 0.18 ± 0.10 mV, respectively. The CV of the DZ was 0.06 ± 0.04 m/s. The total number of DZs per chamber was 1.1 ± 0.3.

**Figure 1:**
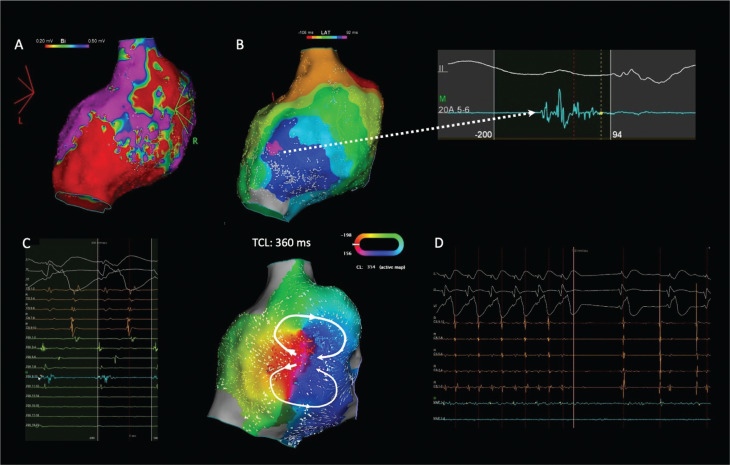
**A:** Right atrial voltage map in a patient with previous right atrial surgery with conduit due to a double-outlet right ventricle demonstrating heterogeneous low-voltage tissue in the lateral wall. **B:** Isochronal late activation mapping demonstrated deceleration zone in the lateral wall with characteristic continuous-fragmented signals (dashed white arrow). The activation map revealed figure-of-eight localized re-entry (white circle) with the critical isthmus **(C)** corresponding to the deceleration zone of isochronal late activation mapping in **B**. **D:** Termination of atrial tachycardia after radiofrequency ablation from the same spot. *Abbreviation:* TCL, tachycardia cycle length.

All DZs related to the CI of inducible ATs were located in the low-voltage zone (<0.5 mV) detected by high-density mapping. **Figure 1** depicts an example of localized re-entry with the CI corresponding to the DZ of ILAM within the low-voltage tissue in the lateral wall in a patient with previous RA surgery with conduit due to a double-outlet right ventricle.

### Electrophysiological characteristics during atrial tachycardia

**Figure 2:**
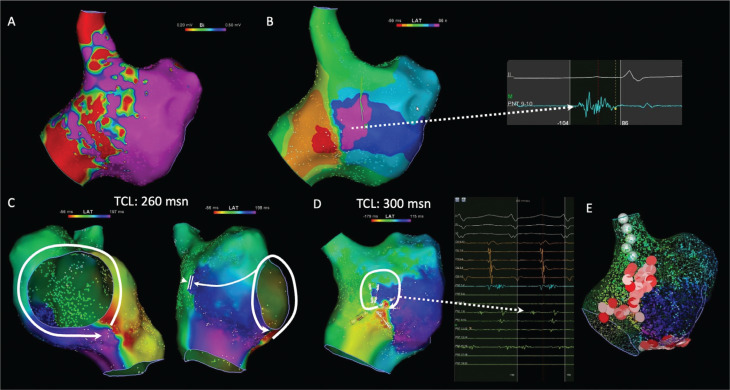
**A:** Right atrial voltage map in a patient without a previous history of ablation demonstrating patchy scar on the lateral wall. **B:** Isochronal late activation mapping during sinus rhythm demonstrated a deceleration zone (white line) on the inferior part of the lateral wall with characteristic continuous-fragmented electrograms (dashed arrow). **C:** Activation map of the atrial tachycardia (cycle length, 260 ms) demonstrated counterclockwise cavotricuspid isthmus (CTI)-dependent macro–re-entry with passive activation of the lateral wall (white circle). After termination of CTI-dependent re-entry, lateral wall localized re-entry was induced with fragmented-continuous activity during atrial tachycardia in the multispline catheter **(D)** coincident with the deceleration zone in **B**. **E:** Ablation lines were created throughout the CTI region as well as the lateral wall, including the whole lateral wall, away from the phrenic nerve simulated site (white tags). *Abbreviation:* TCL, tachycardia cycle length.

In 4/24 (16.7%) patients, CTI-dependent AT was the sole mechanism, whereas non–CTI-dependent re-entry was the mechanism in 9/24 (37.5%) patients. In non–CTI-dependent re-entry, the lateral wall was the most common location of re-entry (70.8%). In 11 (45.8%) patients, >1 ATs were inducible with programmed stimulation. In 8 of these 11 patients, non–CTI-dependent re-entry was inducible after termination of CTI-dependent tachycardia. In all these eight patients, lateral/posterolateral had passive activation during CTI-dependent tachycardia. **Figure 2** demonstrates an example of a DZ in the low-voltage region on the lateral right atrial wall having counterclockwise CTI-dependent macro–re-entry as the first induced AT with bystander activation of the lateral wall. Following termination of CTI-dependent macro–re-entry, programmed stimulation induced lateral wall re-entry with the CI co-localized with the DZ detected during sinus rhythm.

In the whole study group, AT was consistently induced from the region of DZs with fragmented EGMs. The median number of mapped points during AT was 2933 (range, 688–10,827). The detailed procedural characteristics of the study population are demonstrated in **Table 2**. A total of 36 ATs were mapped in the RA (16 [44.4%] localized re-entry, 20 [55.6%] macro–re-entry). The mean EGM duration at CI was 107.9 ± 33.3 ms, and the mean bipolar voltage was 0.19 ± 0.12 mV, with a CV of 0.10 ± 0.08 m/s.

**Table 2:** Procedural Characteristics of the Study Population (n = 24)

Number of mapped ATs, mean, n	1.5 ± 0.7
Mapping system, n (%)	
CARTO™	16 (66.7%)
EnSite™ NavX	8 (33.3%)
Type of anesthesia during catheter ablation, n (%)	
General anesthesia	23 (95.8%)
Conscious sedation	1 (4.2%)
Number of ATs per patient, n (%)	
1 AT	13 (54.2%)
>1 AT	11 (45.8%)
TCL of mapped ATs, mean, ms	303.8 ± 60.9
Number of mapped points, median, n (min–max)	
During FSM mapping	2157 (340–3871)
During tachycardia	2933 (688–10,827)
Characteristics of AT mechanisms, n (%)	36
Localized re-entry	16 (44.4%)
Macro–re-entry	20 (55.6%)
CTI-dependent	13 (36.1%)
Incisional	3 (8.3%)
Septal	1 (2.8%)
Upper loop re-entry	2 (5.6%)
RA dual-loop re-entry	1 (2.8%)
Electrophysiological characteristics during sinus/paced rhythm	
EGM duration, mean, ms	121.7 ± 29.4
Bipolar voltage, mean, mV	0.18 ± 0.10
CV for the DZ, mean, m/s	0.06 ± 0.04
Electrophysiological characteristics of CI during AT	
EGM duration, mean, ms	107.9 ± 33.3
Bipolar voltage, mean, mV	0.19 ± 0.12
CV of CI, mean, m/s	0.10 ± 0.08
Mean follow-up, months	11.7 ± 8.1

*Abbreviations:* AT, atrial tachycardia; CI, critical isthmus; CTI, cavotricuspid isthmus; CV, conduction velocity; EGM, electrogram; FSM, functional substrate mapping; LAT, local activation time; RA, right atrium; TCL, tachycardia cycle length.

### Ablation approach

**Figure 3:**
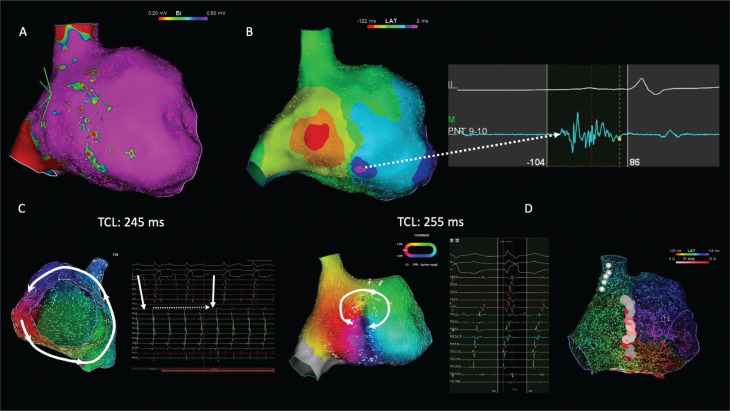
**A:** Right atrial voltage map in a patient with a previous history of tetralogy of Fallot, demonstrating patchy scar on the lateral wall. **B:** Isochronal late activation mapping during sinus rhythm demonstrated a deceleration zone on the inferior part of the lateral wall with characteristic continuous-fragmented electrograms (dashed arrow). **C:** The activation map of the atrial tachycardia (cycle length, 245 ms) demonstrated counterclockwise cavotricuspid isthmus (CTI)-dependent macro–re-entry with clockwise localized re-entry on the lateral wall (white circle). During ablation of the CTI, a 10-ms increase in the tachycardia cycle length and change in the atrial activation sequence were observed (white arrows). **D:** Ablation lines were created throughout the CTI region as well as the lateral wall, away from the phrenic nerve simulated site (white tags). *Abbreviation:* TCL, tachycardia cycle length.

In all patients, the CI of induced AT was targeted, which resulted in 100% termination of AT or a change in the cycle length of the re-entry. In 12 (50%) patients, the initial CTI line terminated AT, while, in the remaining 3 (12.5%) patients, CTI caused a change in either the TCL or the activation sequence. In 19 (79.2%) patients, a linear lateral line from the DZ area to the inferior vena cava was needed to terminate the AT. In all these patients, successful ablation sites in the lateral wall corresponded to DZs **(Figure 3)** **(Video 1)**. A prophylactic lateral line was added to the procedure in five (20.8%) patients with DZs in the lateral wall. In addition, a prophylactic CTI line was created in five (20.8%) patients. Procedure-related complications, including phrenic nerve palsy, pericardial effusion/tamponade, and vascular access site complications, were not observed in any patient.

**Video 1:** The right panel demonstrating the propagation of right atrial ILAM, which revealed late activation with characteristic fragmented-continuous EGM morphology. The left panel demonstrated double loop reentry with counter-clockwise reentry around tricuspid annulus as well as clockwise reentry on the lateral wall sharing the same isthmus in the lateral wall. The critical isthmus on the lateral wall colocalized with the DZ demonstrated on the right panel.

### Correlation between deceleration zones and critical isthmus of atrial tachycardia

In all 24 patients, DZs with characteristic fragmented-continuous EGM morphology were present in the lateral/posterolateral wall. In 19/24 patients with DZs in the lateral wall, CIs of ATs (7/24 with only lateral wall re-entry, 2/24 with upper loop re-entry, and 1/24 with double-loop re-entry, with 9/24 patients having multiple ATs) were co-localized, with DZs detected during FSM. Among these, continuous-fragmented activity was detected during sinus/paced rhythm at the successful ablation site in all patients.

**Figure 4:**
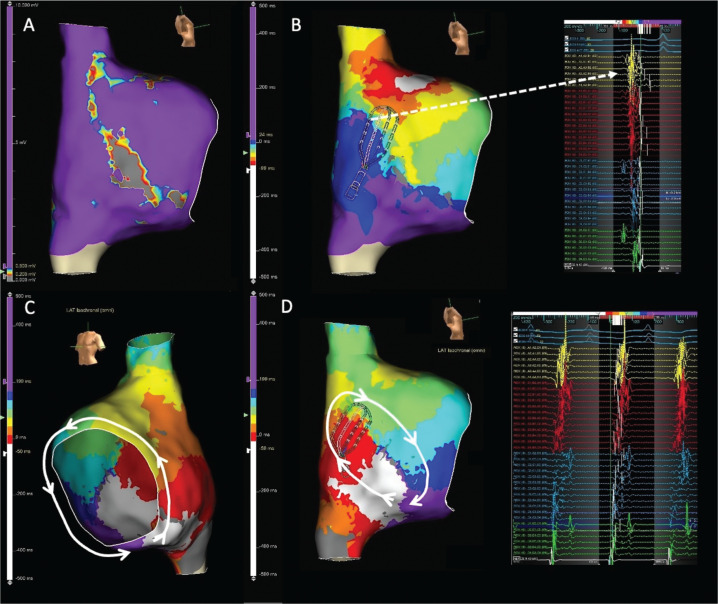
**A:** Right atrial voltage map (0.1–0.5 mV) in a patient with previous atrial septal defect operation demonstrating a linear low-voltage zone in the right atrial lateral wall. **B:** Using a high-density grid catheter, isochronal late activation mapping revealed deceleration zones with fragmented electrogram morphology on the lateral wall (dashed arrows). The activation map of the atrial tachycardia (cycle length, 265 ms) revealed multiple-loop re-entry, including counterclockwise cavotricuspid isthmus–dependent macro–re-entry **(C)** and clockwise lateral wall re-entry (white circle) **(D)**. The critical isthmus of lateral wall re-entry was co-localized with the deceleration zone during sinus rhythm shown in **B**.

Among all ATs, 13/36 (36.1%) were CTI-dependent ATs, and, as the CTI region is the collusion site of sinus or high RA pacing, a DZ was not detected in this region. Furthermore, in five (20.8%) patients with FSM during CS pacing, DZs were still not detected in the CTI region. In all localized re-entries (16/36), DZs in the lateral/posterolateral wall totally correlated with the CI of re-entry. For non–CTI-dependent macro–re-entries (7/36), the lateral wall was the most common site for DZs (three incisional lateral walls, one dual-loop, and two upper loop re-entry). In one patient with interatrial septal re-entry, a DZ was detected in the septal region. **Figure 4** demonstrates a case of multiple-loop re-entry in a patient with a DZ in the lateral wall. In addition to CTI-dependent macro–re-entry, a CI of lateral wall re-entry was detected in the same region as a DZ during sinus rhythm.

In two patients, >1 DZs were detected. Among a total of 26 DZs, 5 (19.2%) were not associated with any inducible ATs during the procedure. The positive predictive value of DZs to detect the CI of inducible ATs was 80.8%.

### Follow-up

**Figure 5:**
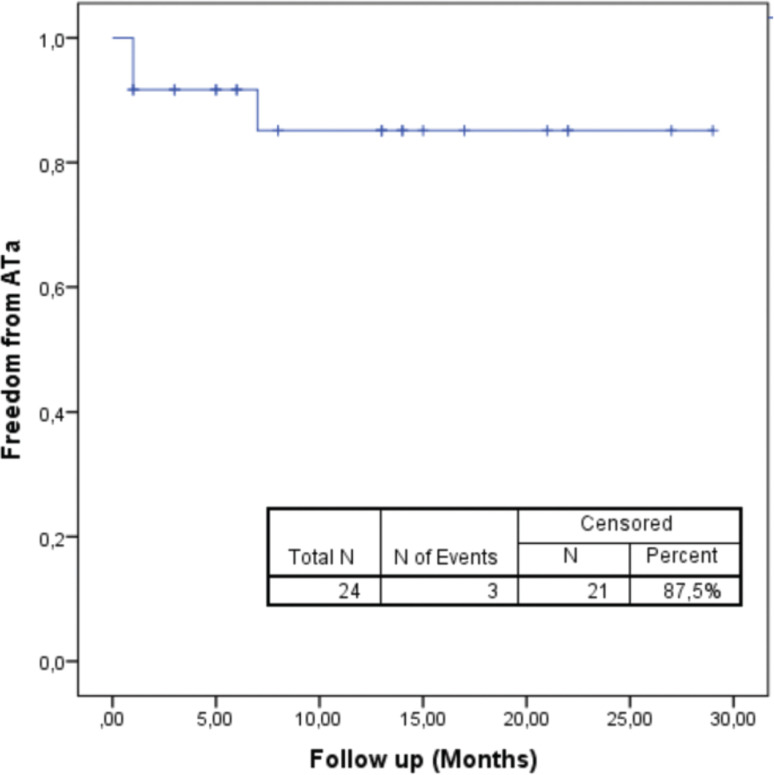
Freedom from atrial tachyarrhythmia after ablation during the mean follow-up of 11.7 ± 8.1 months.

During a mean follow-up of 11.7 ± 8.1 months, the rate of freedom from ATa was 87.5% **(Figure 5)**. In the first 3 months after ablation, five (20.8%) patients were on AAD therapy, whereas three (12.5%) patients were on AADs for >3 months postablation. Among three (12.5%) patients with ATa recurrences, AT was identified in two (8.4%), whereas one (4.2%) had AF. In one patient with AT recurrence, left AT was detected in the redo procedure.

## Discussion

Our study findings demonstrated that FSM during sinus/paced rhythm could reveal relevant data to predict potential critical sites of re-entry in RA among patients with baseline atrial scar. Although the main mechanism of AT was CTI-dependent atrial flutter, DZs detected by ILAM during sinus/paced rhythm successfully predicted the CI of the second most common AT—lateral wall re-entry—in such patients. In DZs, continuous-fragmented EGM was the characteristic signal morphology, which corresponded well with the CI during AT. Our intermediate-term outcomes revealed favorable outcomes of 87% freedom from ATa, compatible with previous data.^2^ These findings highlight the significance of FSM in patients with a right atrial scar to predict ATs beyond CTI-dependent flutter.

The role of the underlying atrial scar as a surrogate to demonstrate slow conduction regions that harbor the CI of re-entry has long been known.^15–17^ Furthermore, several previous studies highlighted the role of substrate characteristics beyond voltage data in terms of DZs during ILAM or decrement-evoked potentials during pacing to predict CI of left atrial re-entry.^5,7,18^ In a multicenter analysis, Woods et al.^7^ demonstrated that DZs during sinus or paced rhythm correlated well with the CI of localized re-entry in left ATs using high-resolution mapping. In addition, Tsai et al.^6^ reported that functional characteristics of the left atrium might identify the CI of re-entrant ATs using high-density mapping during sinus rhythm. In a recent study, we corroborated the previous data in terms of the utility of FSM during sinus rhythm to predict the CI of ATs among patients with left atrial low-voltage zones.^8^ However, functional substrate characteristics of the RA during sinus rhythm beyond voltage data were not evaluated before. Due to the potential role of FSM in addition to voltage mapping, we aimed to elucidate functional characteristics of RA that might have a role in the maintenance of ATs.^19^

In patients with structurally normal hearts, CTI-dependent atrial flutter is the most commonly reported mechanism of AT in RA.^20^ Moreover, CTI-dependent atrial flutter is still the most common form of AT in patients with low-voltage areas in RA due to congenital heart disease or previous right atrial surgery.^21^ The change in atrial conduction properties due to volume/pressure overload as a result of valvular dysfunction, surgical incisions, or fibrosis due to several congenital cardiac defects may lead to both CTI-dependent and non–CTI-dependent macro–re-entry.^1,2,22^ Right atrial cannulation during surgery or surgical incisions may specifically create the substrate for non–CTI-dependent re-entry, especially in the lateral and posterolateral walls.^2^ As a result, multiple ATs can be observed in such patients.

The characteristics of the re-entrant circuit in patients with incisional macro–re-entry have been extensively studied before. In a previous study by Nakagawa et al.,^23^ the EGM exhibited single, double, or fragmented atrial potentials in termination sites of critical channel with extremely low (<0.1 mV) bipolar amplitude. Similar to these findings, our data revealed a low bipolar voltage amplitude of 0.23<nonbrfixspace>±<nonbrfixspace>0.17 mV during sinus rhythm and 0.26<nonbrfixspace>±<nonbrfixspace>0.19 mV during AT, harboring the DZs during sinus rhythm and the CI of re-entry, respectively. Despite the high acute procedural success, ablation of clinically presenting AT is associated with a significant rate of recurrences despite successful termination in the case of underlying atrial scar.^24^ Therefore, aiming all potential sites of re-entry in the index procedure targeting both scar-related regions in addition to classic isthmuses appears to have favorable long-term freedom from arrhythmias.^25^

Although the lateral/posterolateral wall is the most common location of non–CTI-dependent re-entry in RA, functional characteristics other than voltage analysis have not been extensively evaluated. Our data imply that the lateral/posterolateral wall harbors DZs in 20/24 patients during sinus/paced rhythm corresponding to the CI of re-entry. On the other hand, a potential limitation of FSM is discrimination between the EGMs belonging to the CI of AT or bystander regions in the low-voltage zones having fragmented-continuous EGM morphology. Although DZs in the lateral wall predicted well the induction of re-entry in eight (33.3%) patients after termination of CTI-dependent macro–re-entry, in five (20.8%) patients, no additional tachycardia was inducible in the lateral wall of the RA despite the presence of DZs. Although these DZs are considered to be bystander regions, an ablation line was created to prevent future recurrences.^25^ Furthermore, when the probability of non-inducibility is considered, targeting these DZs, rather than classifying them as bystander regions, may be beneficial. Whether an ablation strategy based on empirical ablation of all DZs in the low-voltage region may decrease future recurrences necessitates further prospective comparative studies.

To the best of our knowledge, this is the first study in the literature evaluating the functional characteristics of RA late activation during sinus/paced rhythm. The main novelty of late activation mapping is the demonstration of slow conduction regions defined as DZs during sinus rhythm. These regions are well correlated with the CI of re-entrant right ATs. In addition, continuous-fragmented EGM morphology is the common finding in both DZs and the CI of re-entry. Despite the availability of recent evidence regarding the association of DZs during sinus rhythm in LA, there is a paucity of data regarding the utility of such a novel mapping approach in the RA. Furthermore, evaluation of these DZs for decremental characteristics during programmed atrial stimulation in terms of decrement-evoked potential mapping would yield insights regarding the actual role of these sites in the maintenance of re-entry. When the high-recurrence rate of right ATs, especially in patients with underlying low-voltage zones, is considered, baseline evaluation and ablation of potential critical sites that may have a role in the maintenance of re-entry can provide better freedom from recurrence. Despite this being a small-scale short-term follow-up study, a high rate of freedom from recurrences may provide insights regarding the potential ablation of these DZs in addition to clinical tachycardia to improve future outcomes.

Our study has several limitations. First, this was a small-scale single-center study including patients referred only for AT ablation; therefore, the generalizability of these findings warrants further large-scale studies. Second, our findings indicated the utility of this method in predicting the CI of re-entrant ATs in patients with baseline atrial scar. Third, entrainment was not performed in all cases when degeneration into another AT/AF or termination of AT was considered. On the other hand, high-density activation maps provided an accurate site of ablation in all patients. Fourth, wavefront collision occurs in the CTI region of RA during sinus/high RA pacing; therefore, functional characteristics of the CTI region could not be determined in the majority of the patients. Finally, the pacing site may affect the CV and intracardiac EGMs due to the change in the direction of the activation wavefront variation.^26^

In conclusion, our findings demonstrated the utility of FSM during sinus/paced rhythm in the RA to predict the critical sites of re-entry. As both DZs and continuous-fragmented EGMs are well correlated with the successful ablation sites of ATs, these areas can be potential ablation targets in the case of non-inducible ATs. Further comparative studies are needed to evaluate the role of atrial FSM to tailor ablation strategy in patients with the right atrial low-voltage substrate.
